# Impact of continuity of care on cardiovascular disease risk among newly-diagnosed hypertension patients

**DOI:** 10.1038/s41598-020-77131-w

**Published:** 2020-11-17

**Authors:** Daein Choi, Seulggie Choi, Hyunho Kim, Kyuwoong Kim, Nakhyun Kim, Ahryoung Ko, Kyae Hyung Kim, Joung Sik Son, Jae Moon Yun, Yoon Kim, Sang Min Park

**Affiliations:** 1 Department of Biomedical Sciences, College of Medicine, Seoul National University Graduate School, Seoul, South Korea; 2 Department of Medicine, Mount Sinai Beth Israel, Icahn School of Medicine at Mount Sinai, New York, USA; 3 Department of Family Medicine, Seoul National University Hospital, Seoul, South Korea; 4 National Cancer Control Institute, National Cancer Center, Goyang, South Korea; 5 Department of Public Health & Medical Service, Seoul National University Hospital, Seoul, South Korea; 6 Department of Health Policy and Management, Seoul National University College of Medicine, Seoul, South Korea; 7 Institute of Health Policy and Management, Medical Research Center, Seoul National University, Seoul, South Korea

**Keywords:** Epidemiology, Cardiology, Cardiovascular diseases, Risk factors

## Abstract

Several previous studies have noted benefits of maintaining continuity of care (COC), including improved patient compliance, decreased health care cost, and decreased incidence of hospitalization. However, the association of COC in hypertension patients with subsequent cardiovascular disease (CVD) risk is yet unclear. Therefore, we aimed to investigate the impact of COC on CVD risk among newly-diagnosed hypertension patients. We conducted a cohort with a study population consisted of 244,187 newly-diagnosed hypertension patients in 2004 from the Korean National Health Insurance Service database. The participants were then divided into approximate quartiles of COC index, and followed from 1 January 2007 until 31 December 2017. Cox proportional hazards models were used to evaluate the adjusted hazard ratios (aHRs) and 95% confidence intervals (CIs) for CVD risk according to quartiles. Compared to patients within the lowest quartile of COC index, those within the highest quartile of COC index had reduced risk for CVD (aHR 0.76, 95% confidence interval; CI 0.73–0.79), CHD (aHR 0.66, 95% CI 0.62–0.69) and stroke (aHR 0.84, 95% CI 0.80–0.88). COC among hypertension patients was associated with improved medication compliance and reduced risk of stroke and CVD. The importance of maintaining COC should be emphasized to reduce the risk of CVD among hypertension patients.

## Introduction

Hypertension is known to be a major modifiable risk factor for cardiovascular disease (CVD)^1^, and it is also one of the leading causes of a preventable death^2^. In 2017, the American College of Cardiology/American Heart Association released an updated guideline. According to the new criteria for hypertension, 46% of the United State adults are affected by high blood pressure^3^. However, approximately 30% of those who affected are not aware of the disease, and less than 60% are adequately controlled worldwide^4^. Since CVD is also recognized as the leading cause of death^5^, it is important to emphasize proper management of hypertension to reduce the risk of CVD and mortality.

Meanwhile, previous studies have noted poor care coordination on chronic diseases has been associated with increase in medical expense^6,7^. Continuity of care (COC), which generally refers to the relationship between patient and their physician overtime^8^, is known to improve quality of care and patient compliance^9,10^. Despite these benefits, not having COC on patients with chronic disease is common, especially in Asian countries where primary care physician is not established commonly^11^. Although it is likely that COC among chronic diseases patients such as hypertension would benefit from their comprehensive care, the impact of COC on clinical outcomes are relatively less studied. Previous studies investigated the effect of COC among hypertension patients focused on the outcomes of blood pressure control^12^, medication compliance^13^, health care cost^7^, and incidence of hospital admission^14^. However, the association of COC with CVD is still unexplored especially among hypertension patients.

Therefore, we aimed to investigate the impact of COC on cardiovascular risk among newly-diagnosed hypertension patients by using nationwide health claim database from Korean National Health Insurance Service (NHIS). Also, we evaluated the association of COC with medication compliance as a secondary outcome as well.

## Methods

### Study population

The study population was derived from NHIS database. Nearly all South Korean citizens are ensured under the NHIS, with an enrollment rate of 97%. The NHIS provides mandatory health care covering most forms of health services, and the data from insured health services including hospital use inpatient and outpatient, pharmaceutic drug prescriptions are collected in the NHIS database^15,16^. Additionally, NHIS provides biannual national health screening examinations for enrollees. The results of health examinations are also collected by the NHIS and provided for research purposes. These data include anthropometric measurements, a self-reported questionnaire on participants’ health behaviors, and laboratory tests for blood^15^. The NHIS database has been used for multiple previous epidemiological studies, and its validity is described detail elsewhere^17,18^.

**Figure 1:**
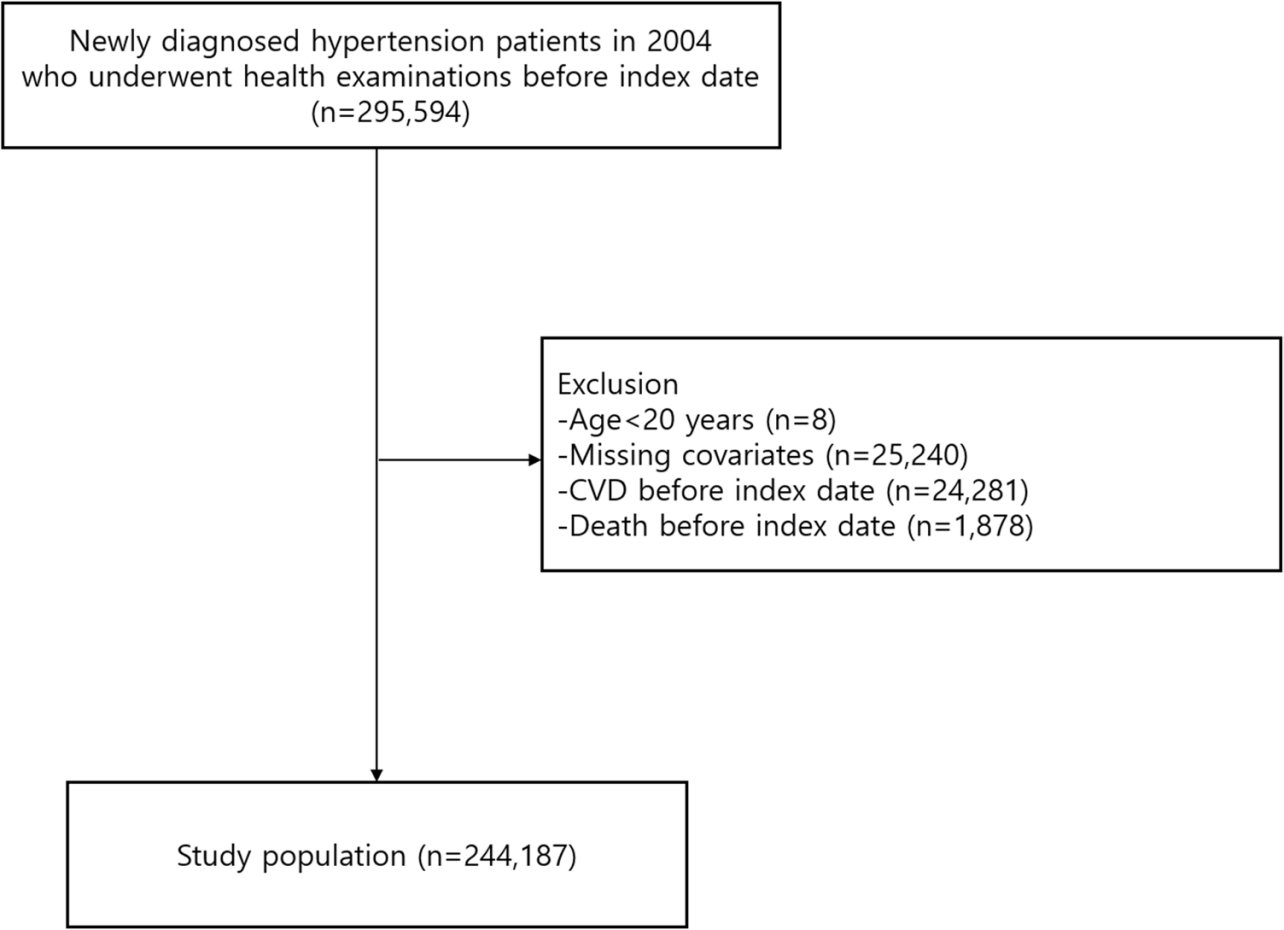
Flow diagram of selection of study participant.

Among 295,594 newly-diagnosed hypertension patients in 2004, we excluded 8 participants who were aged less than 20 years. Then, 25,240 participants with missing values for covariates were further excluded. Finally, 1878 and 24,281 participants who died or diagnosed with CVD prior to the index date of 1 January 2007 were excluded, respectively. The final study population consisted of 244,187 participants who underwent health examinations before index date. All participants were followed-up from 1 January 2007 until the event of CVD, death, or 31 December 2017, whichever came earliest. Figure 1 presents the flow diagram of selecting study participants.

### Key variables

Hypertension was defined as the prescription of anti-hypertensive medication under the diagnosis code for hypertension (I10) under the International Classification of Diseases Tenth Edition (ICD-10). Participants with less than 4 visits within the first 2 years of diagnosis were not included in the newly-diagnosed hypertension patients. The rationale of this exclusion criteria was based on previous studies that investigated the COC^19,20^. It was noted that continuity cannot be assessed well with few visits, since it’s relatively easy to attain its maximum (1) or minimum (0) value with few visits^19^. Previous studies have adopted 2-year exposure periods to ensure longitudinal continuity^20^, and analyzed participants who had 4 or more visits^19^. COC was measured by the COC index and the number of medical institutions utilized^21,22^. COC index is a widely used measure of continuity that reflects both the frequency of visits to each provider and the dispersion of visits between providers^23,24^. Hospital use for hypertensive medication prescriptions in South Korea requires the medical records for Korean National Health Insurance reimbursement. Therefore, almost every hospital uses including outpatient and inpatient for hypertension is recorded in NHIS, which makes the COC index calculated by claim data of NHIS valid and accurate. COC index and the number of medical institutions a person visited within first 2 year of diagnosis, which was 2005–2006, according to all outpatient visit. The participants were then divided into approximate quartiles, with participants among the first quartile having lowest COC index or the number of medical institutions utilized.

The event of CVD was defined when a participant was hospitalized more than 2 days under diagnosis code for CHD (ICD-10 codes for I20-I21) or stroke (ICD-10 codes for I60-I69)^25^. Medication compliance was determined by medication possession ratio (MPR). MPR was calculated by the proportion of the hypertensive medication prescriptions days during the first 2 years of follow-up period (2007–2008). High medication compliance was defined when a participant had an MPR value of 0.8 or higher^26,27^.

The considered covariates included age (continuous, years), sex (categorical, men and women), household income (categorical, 1st , 2nd , 3rd , and 4th quartiles), smoking status (categorical, never, past, and current smokers) alcohol consumption (categorical, 0, 0–1, 1–2, 3–4, and 5 or more times per week), physical activity (categorical, 0, 1–2, 3–4, 5–6, and 7 times per week), body mass index (continuous, kg/m^2^), fasting serum glucose (continuous, mg/dL), and Charlson comorbidity index (continuous). Smoking status, alcohol consumption and physical activity were assessed by a self-reported questionnaire during the health examination. Household income was derived from the insurance premium and the Charlson comorbidity index was calculated with an algorithm adopted form a previous study^28,29^.

### Statistical analysis

**Figure 2:**
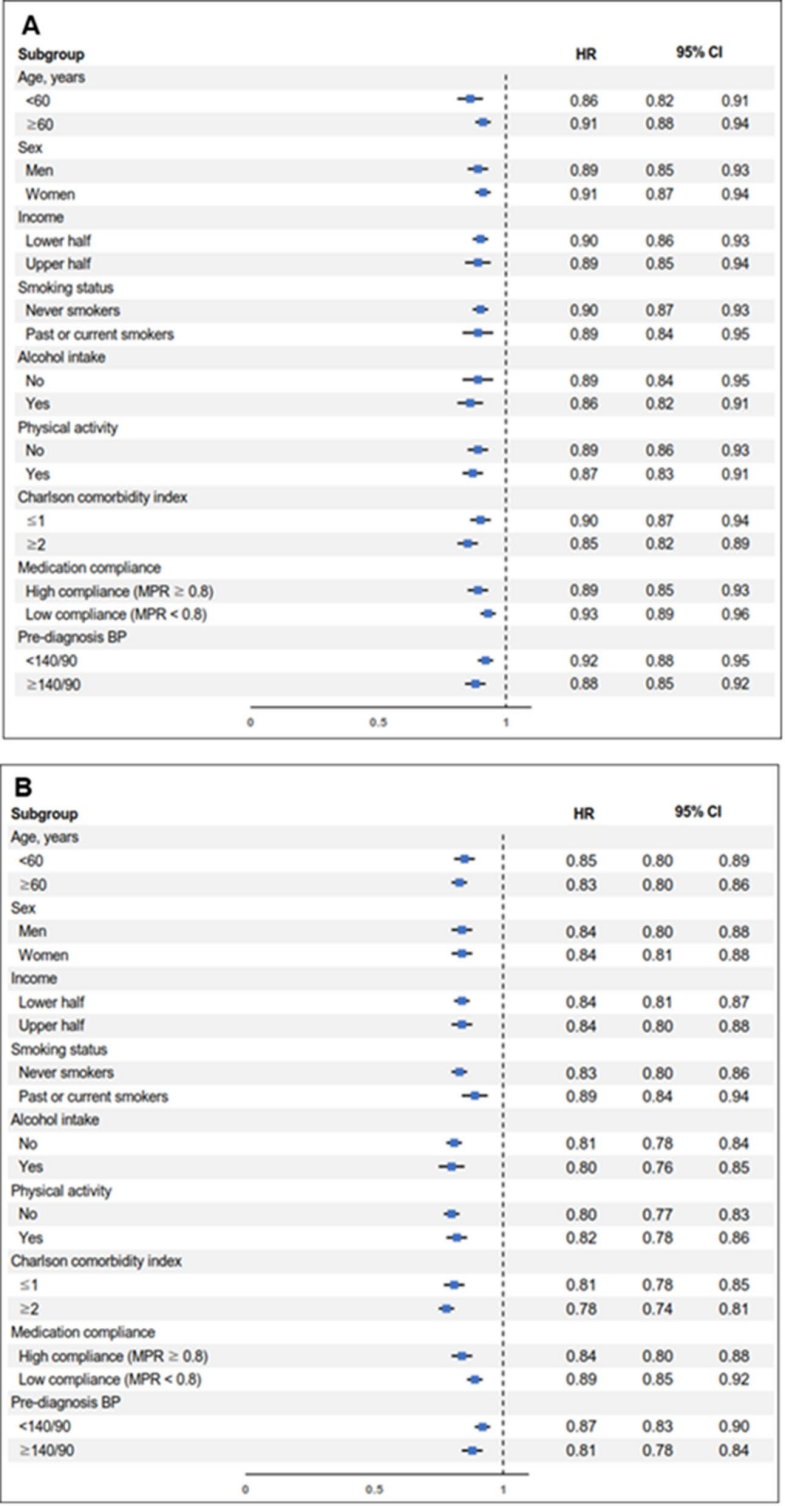

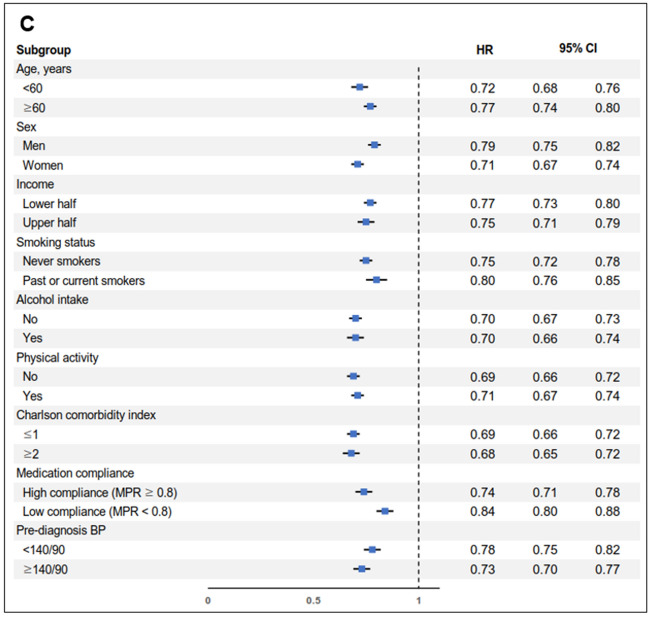
Stratified analysis on the association of continuity of care with cardiovascular disease according to subgroups of age, smoking, alcohol intake, physical activity, Charlson comorbidity index, and medication compliance. (**A**) Adjusted hazard ratio for cardiovascular disease of participants in 2nd quartile compared to those of 1st quartile. (**B**) Adjusted hazard ratio for cardiovascular disease of participants in 3rd quartile compared to those of 1st quartile. (**C**) Adjusted hazard ratio for cardiovascular disease of participants in 4th quartile compared to those of 1st quartile. *HR* adjusted hazard ratio, *CI* confidence interval, *MPR* medication possession ratio, *BP* blood pressure.

Chi-square test for categorical variables and analysis of variance for continuous variables were conducted to compare the difference in distributions of covariates according to the quartiles of COC index. Multivariate Cox proportional hazard regression analyses were conducted to evaluated adjusted hazard ratios (aHRs) and 95% confidence intervals (CIs) for CVD, CHR and stroke risk according to the COC index and the number of medical institutions utilization quartile groups. The proportionality assumption of the Cox proportional hazards regression has been visually tested and validated using the Schoenfeld residual method. We also conducted multivariate logistic regression to determine the adjusted odds ratios (aORs) and 95% CIs for hypertensive medication compliance according to the quartiles of COC index. Stratified analyses on the association of COC index with CVD were conducted according to the subgroups of age, sex, smoking status, alcohol consumption, physical activity, Charlson comorbidity index, medication compliance, and pre-diagnosis blood pressure. (Fig. 2) Additionally, analyses using the usual provider continuity (UPC) score and the modified modified continuity index (MMCI) as other measures of COC were conducted^21^. The methods of calculating theses indices are well established and noted in multiple previous studies^23,24,30,31^.

Statistical significance was defined as a two-side *p* value of less than 0.05. All data collection and statistical analyses were conducted using SAS Enterprise Guide 7.1 (SAS Institute, Cary, USA).

### Ethical considerations

This study was approved by the Seoul National University Hospital Institutional Review Board (IRB number: E-1812-041-993) and the analyses were performed in accordance with relevant guidelines and regulations. The requirement for informed consent was waived as the NHIS database was constructed after anonymization according to strict confidentiality guidelines.

## Results

The descriptive characteristics of the study population according to COC index quartiles are depicted in Table 1. The range of COC index were 0.00–0.23, 0.23–0.36, 0.36–0.56, and 0.57–1.00 for the 1st, 2nd, 3rd, and 4th quartiles groups. Participants with higher COC index tended to be young age, men, have lower household income, smoke often, consume more alcohol, had higher pre-diagnosis blood pressure, obese, and have less comorbidities.

**Table 1:** Descriptive characteristics of the study population.

	Continuity of care index, quartiles				*p* value
	1st (lowest)	2nd	3rd	4th (highest)	
Range	0.00–0.23	0.23–0.36	0.36–0.55	0.55–1.00	
N	60,960	61,080	61,080	61,067	
Age, years, mean (SD)	60.7 (10.7)	59.8 (10.7)	58.6 (10.8)	56.8 (10.8)	< 0.001
**Sex, N (%)**					< 0.001
Men	24,031 (39.4)	28,273 (46.3)	32,734 (53.6)	39,012 (63.9)	
Women	36,929 (60.6)	32,807 (53.7)	28,346 (46.4)	22,055 (36.1)	
**Household income, quartiles, N (%)**					< 0.001
1st (highest)	23,715 (38.9)	22,767 (37.3)	22,344 (36.6)	21,707 (35.6)	
2nd	14,492 (23.8)	14,827 (24.3)	14,773 (24.3)	15,016 (24.6)	
3rd	10,160 (16.7)	10,607 (17.2)	10,967 (18.0)	11,119 (18.2)	
4th (lowest)	12,593 (20.7)	12,979 (21.3)	12,996 (21.3)	13,225 (21.7)	
**Smoking status, N (%)**					< 0.001
Never smoker	48,982 (80.4)	46,511 (76.2)	43,690 (71.5)	39,062 (64.0)	
Past smoker	5038 (8.3)	5709 (9.4)	6547 (10.7)	7247 (11.9)	
Current smoker	6940 (11.4)	8860 (14.5)	10,843 (17.8)	14,758 (24.2)	
**Alcohol intake, times per week, N (%)**					< 0.001
0	42,777 (70.2)	39,909 (65.3)	36,366 (59.5)	31,547 (51.7)	
0–1	6396 (10.5)	6936 (11.4)	77,670 (12.6)	8,454 (13.8)	
1–2	6830 (11.2)	8162 (13.4)	9781 (16.0)	12,293 (20.1)	
3–4	2957 (4.9)	3570 (5.8)	4494 (7.4)	5593 (9.2)	
5 or more	2000 (3.3)	2503 (4.1)	2769 (4.5)	3180 (5.2)	
**Physical activity, times per week, N (%)**					
0	33,764 (55.4)	32,938 (53.9)	31,873 (52.2)	30,144 (49.4)	< 0.001
1–2	12,746 (20.9)	13,472 (22.1)	14,441 (23.6)	16,412 (26.9)	
3–4	6704 (11.0)	6826 (11.2)	7049 (11.5)	7266 11.9)	
5–6	1705 (2.8)	1800 (3.0)	2026 (3.3)	1835 (3.0)	
7	6041 (9.9)	6044 (9.9)	5691 (9.3)	5410 (8.9)	
**Charlson comorbidity index, N (%)**					
≤ 1	30,168 (49.5)	36,351 (59.5)	41,204 (67.5)	46,823 (76.7)	< 0.001
2	13,031 (21.4)	11,868 (19.4)	10,286 (16.8)	8035 (13.2)	
3	8199 (13.5)	6499 (10.6)	5240 (8.6)	3688 (6.0)	
≥ 4	9562 (15.7)	6362 (10.4)	4350 (7.1)	2521 (4.1)	
**Pre-diagnosis BP**					
< 140/90	37,148 (53.4)	36,286 (52.2)	35,666 (51.3)	34,171 (49.1)	< 0.001
≥ 140/90	32,414 (46.6)	33,276 (47.8)	33,896 (48.7)	35,391 (50.9)	
**BMI**					
< 23.0	17,917 (25.8)	17,482 (25.1)	17,127 (24.6)	16,815 (24.2)	< 0.001
23.0–24.9	18,473 (26.6)	18,318 (26.3)	18,055 (26.0)	17,833 (25.6)	
≥ 25.0	33,172 (47.7)	33,762 (48.5)	34,380 (49.4)	34,914 (50.2)	
DM prevalence (%)	16,558 (23.8)	15,805 (22.7)	14,848 (21.3)	13,531 (19.5)	< 0.001
CKD prevalence (%)	472 (0.7)	447 (0.6)	351 (0.5)	313 (0.5)	< 0.001

*p* value calculated by Chi squared test for categorical variables and analysis of variance for continuous variables.

*N* number of people, *SD* standard deviation.

Table 2 shows the risk of CVD, CHD, and stroke according to quartiles of the COC index. Compared to the participants with the lowest COC index, those with the highest COC index had 24% lower risk for CVD, 34% lower risk for CHD, and 15% lower risk for stroke. Furthermore, the risk reduction of CVD, CHD, and stroke tended to be higher according to the higher quartiles of the COC index (all *p* for trend < 0.001).

**Table 2:** Hazard ratios for cardiovascular disease according to continuity of care index.

	Continuity of care index, quartiles				*p* for trend
	1st (lowest)	2nd	3rd	4th (highest)	
Range	0.00–0.23	0.23–0.36	0.36–0.56	0.57–1.00	
N	60,960	61,080	61,080	61,067	
**Cardiovascular disease**					
Events	9827	8610	7767	6718	
Person-years	580,052	590,626	598,593	608,375	
aHR (95% CI)	1.00 (reference)	0.90 (0.87–0.92)	0.84 (0.81–0.87)	0.76 (0.73–0.78)	< 0.001
**Coronary heart disease**					
Events	4571	3917	3485	2938	
Person-years	580,052	590,626	598,593	608,375	
aHR (95% CI)	1.00 (reference)	0.86 (0.82–0.90)	0.77 (0.74–0.81)	0.66 (0.62–0.69)	< 0.001
**Stroke**					
Events	5256	4693	4282	3780	
Person-years	580,052	590,626	598,593	608,375	
aHR (95% CI)	1.00 (reference)	0.93 (0.89–0.96)	0.90 (0.86–0.94)	0.85 (0.81–0.89)	< 0.001

Hazard ratio calculated by Cox proportional hazards regression after adjustments for age, sex, household income, smoking status, alcohol intake, physical activity, Charlson comorbidity index, body mass index, and fasting serum glucose.

*N* number of people, *aHR* adjusted hazard ratio, *CI* confidence interval.

Similarly, Table 3 depicts the risk of CVD, CHD, and stroke according to the number of medical institutions utilized. Participants who visited the greatest number of medical institutions (> 9 institutions) had 33% increased risk for CVD, 63% increased risk for CHD, and 14% increased risk for stroke, compared to those with the least number of medical institutions (1–3 institutions). As the number of medical institutions utilized increased, statistically significant increased risk of developing CVD, CHD, and stroke were observed (all *p* for trend < 0.001).

**Table 3:** Hazard ratios for cardiovascular disease according to the number of medical institutions utilized.

	The number of medical institutions utilized, quartiles				*p* for trend
	1st (lowest)	2nd	3rd	4th (highest)	
Range	1–3	4–5	6–8	9–76	
N	51,657	57,827	70,527	64,176	
**Cardiovascular disease**					
Events	5704	7152	9541	10,525	
Person-years	512,832	568,934	686,446	609,434	
aHR (95% CI)	1.00 (reference)	1.08 (1.05–1.12)	1.16 (1.12–1.20)	1.33 (1.29–1.38)	< 0.001
**Coronary heart disease**					
Events	2456	3112	4359	4984	
Person-years	512,832	568,934	686,446	609,434	
aHR (95% CI)	1.00 (reference)	1.13 (10.08–1.29)	1.31 (1.24–1.38)	1.63 (1.54–1.71)	< 0.001
**Stroke**					
Events	3248	4040	5182	5541	
Person-years	512,832	568,934	686,446	609,434	
aHR (95% CI)	1.00 (reference)	1.05 (0.99–1.10)	1.05 (1.00–1.10)	1.14 (1.08–1.19)	< 0.001

Hazard ratio calculated by Cox proportional hazards regression after adjustments for age, sex, household income, smoking status, alcohol intake, physical activity, Charlson comorbidity index, body mass index, and fasting serum glucose.

*N* number of people, *aHR* adjusted hazard ratio, *CI* confidence interval.

The association of COC with hypertensive medication compliance is shown in Table 4. While 44.8% of participants with the lowest COC index maintained high medication compliance of MPR more than 0.8, 61.9% of those with the highest COC index maintained high medication compliance among the first 2 years of hypertension diagnosis. Compared to the patients with the lowest COC, those with the highest COC had higher odds (aOR 1.76, 95% CI 1.71–1.79) for having high medication compliance.

**Table 4:** Association of continuity of care with hypertensive medication compliance.

	Continuity of care index, quartiles				*p* for trend
	1st (lowest)	2nd	3rd	4th (highest)	
N	60,960	61,080	61,080	61,067	
**High medication compliance**					
Cases (%)	27,309 (44.8)	31,707 (51.9)	34,568 (56.7)	37,788 (61.9)	
aOR (95% CI)	1.00 (reference)	1.27 (1.24–1.30)	1.47 (1.44–1.50)	1.76 (1.71–1.79)	< 0.001

High medication compliance determined as medication possession ratio of 0.8 or higher during the first 2 years of follow-up.

Odds ratio calculated by logistic regression after adjustments for age, sex, household income, smoking status, alcohol intake, physical activity, Charlson comorbidity index, body mass index, and fasting serum glucose.

*N* number of people, *aOR* adjusted odds ratio, *CI* confidence interval.

Figure 2 shows the results from stratified analyses of the impact of COC on CVD according to the subgroups of age, sex, smoking status, alcohol consumption, physical activity, Charlson comorbidity index, medication compliance, and pre-diagnosis blood pressure. The risk reduction of CVD among the high COC index group was preserved among the subgroups mentioned above, particularly regardless of medication compliance. This result from the stratified analysis demonstrates that COC has a benefit in reducing the risk of CVD independent of medication compliance.

The risk of CVD, CHD, and stroke according to other measures of COC is shown in Supplementary Table S1. The results were consistent with those of Table 2, showing that maintaining higher UPC score and MMCI was associated with reduced risk for CVD, CHD and stroke.

## Discussion

In this nationwide population-based study among 244,187 newly-diagnosed hypertension patients, we have shown that COC was associated with reduced risk of CVD. We have also applied an intuitive measure of COC, the number of medical institutions utilized, to evaluate the CVD risk associated with COC. The increasing number of utilized medical institutes, which represents lower COC, was associated with a higher risk for CVD compared to those who visited fewer hospitals. Furthermore, we have demonstrated that higher COC was associated with higher medication compliance. While several previous studies have investigated the benefits of COC among hypertension patients^7,12–14^, none of these focused on the risk of developing CVD.

Several possible mechanisms could explain the association of COC with decreased risk of cardiovascular diseases observed in this study. It is noted that higher COC among hypertension patients is associated with higher health-related quality of life^32^, which reflects both physical function and mental health. Likewise, patients with high COC are more likely to receive a higher quality of care and improve lifestyle behaviors as noted in the previous studies^9,33^. Moreover, patients with higher COC are associated with a higher level of trust in physicians along with higher patient satisfaction^34,35^, which is directly linked to medication compliance as shown in this study as well^36,37^. Particularly among hypertension patients, some studies noted that the higher COC is also associated with the higher rate of controlled blood pressure^12^, which is also directly associated with the risk reduction of cardiovascular disease.

Previous studies have evaluated the positive effect of COC on the concept of health care expense. These studies reported that maintaining COC is associated with the decreased number of emergency department visits^24^, hospitalizations^10^, and health care costs^7,24^. Other studies investigated the effect of COC among chronic diseases mainly focused on diabetic patients. These studies demonstrated that higher COC is associated with medication adherence^31^, better glycemic control^37^, and decreased mortality^22,38^. Similarly, a few recent studies evaluated the direct impact of COC on clinical outcomes, mostly focused on the mortality^39–41^.

Although numerous studies noted the benefits of maintaining COC, relatively few studies evaluated the effect of COC among hypertension patients. McClellan and colleagues evaluated 4688 hypertension patients and reported that COC was associated with better blood pressure control^12^. In 2011, a study investigated 5590 individuals aged more than 67 years and noted that COC may not increase adherence to hypertensive medication. Most recently, Ye and colleagues demonstrated higher COC among hypertension patients improved health-related quality of life^32^, and Nam and his colleagues reported the association of higher COC and decreased the risk of hospital admission in hypertension patients. However, the association of COC with the risk of disease development among hypertension patients was not noted in these studies. The result of this study further expands the concept of previous studies and demonstrated that COC is associated with reduced risk of CVD among newly-diagnosed hypertension patients.

There are several limitations to be considered in this study. First, COC was only measured by the pattern of clinical visits, which could not reflect some aspects of COC such as the interpersonal relationship between patient and physicians^21,30^. COC after the index date was not followed and therefore the effect of possible changes was not considered. Third, the study population was restricted to those newly-diagnosed hypertension patients. Hence, long-term complications of hypertension might be underestimated under eleven years of follow-up. Also, other possible complications of hypertension such as heart failure, hypertensive nephropathy, and hypertensive retinopathy were not assessed. Therefore, future studies with a longer follow-up period and assessment of various complications of hypertension are merited. Finally, hypertension medications or dietary intake that participants used were not reflected in our analysis. These factors indirectly reflect the severity of hypertension and might be associated with subsequent CVD. Although we tried to take into account the grade of hypertension and conducted the stratified analysis based on pre-diagnosis blood pressure (Fig. 2), further studies with considering the medications or dietary intake to control hypertension will be merited.

In spite of the limitations mentioned above, our study has some advantages. First, we investigated a large number of study population adjusting a wide range of covariates, which has not done previously. Extensive subgroup analyses of potential confounders also enhance the reliability of our results. Particularly, higher COC was associated with decreased risk of CVD even among those with high medication compliance, implying that COC has a benefit in reducing the risk of CVD independent of medication compliance. Second, we took account for multiple commonly used different measures of COC which led the similar results. Especially, the number of utilized medical institutes, which is an intuitive and simple measure reflects the non-continuity of care also demonstrated our main result effectively. Although the number of medical institutes utilized is an easier way to reflect COC, it has a limitation that it might be associated with severity and progression of hypertension, and therefore further studies on this index will be merited as well. Third, we attempted to clarify the possible mechanisms for the association of COC and reduced CVD risk by investigating medication compliance as an intermediate outcome.

In conclusion, we have shown that the COC was associated with decreased risk for CVD, CHD, and stroke among newly-diagnosed hypertension patients. Higher COC was also associated with improved hypertensive medication compliance. Therefore, the importance of COC should be emphasized to reduce the risk of cardiovascular complications of hypertension.

## Supplementary information

Supplementary Information

## Data Availability

This study is based on Korean National Health Insurance Service Database. These data do not belong to the authors but to the Korean National Health Insurance Service, and the authors are not permitted to share them, except in aggregate form.
