# Supplementary material for: Impact of continuity of care on cardiovascular disease risk among newly-diagnosed hypertension patients
Source: Sci Rep. 2020 Nov 17;10:19991. doi: 10.1038/s41598-020-77131-w (PMC7672066; doi:10.1038/s41598-020-77131-w)
Supplement: Supplementary file 1 — Supplementary Information [file 41598_2020_77131_MOESM1_ESM.docx]

**Impact of Continuity of Care on Cardiovascular Disease risk among Newly-diagnosed Hypertension Patients**

Daein Choi, MD^1,2^; Seulggie Choi, MD^1^; Hyunho Kim^3^, MD; Kyuwoong Kim^1^, BSc; Nakhyun Kim^3^, MD; Ahryoung Ko^3^, MD; Kyae Hyung Kim^3^, MD; Joung Sik Son^3^, MD, MSc; Jae Moon Yun^3^, MD, MPH; Yoon Kim^4^, MD, PhD; Sang Min Park^1,3^ MD, MPH, PhD

^1^Department of Biomedical Sciences, Seoul National University Graduate School, Seoul, South Korea

^2^Department of Medicine, Mount Sinai Beth Israel, Icahn School of Medicine at Mount Sinai, New York, USA

^3^Department of Family Medicine, Seoul National University Hospital, Seoul, South Korea

^4^Department of Health Policy and Management, Seoul National University College of Medicine, Seoul, South Korea

**Supplemental Table 1.** Hazard ratios for cardiovascular disease according to usual provider continuity score and modified modified continuity index.

| **Usual provider continuity score, quartiles** | | | | | *p* for trend |
| --- | --- | --- | --- | --- | --- |
|  | **1^st^ (lowest)** | **2^nd^** | **3^rd^** | **4^th^ (highest)** |  |
| Range | 0.08-0.40 | 0.40-0.55 | 0.55-0.73 | 0.74-1.00 |  |
| N | 60,006 | 62,094 | 60,991 | 61,047 |  |
| Cardiovascular disease |  |  |  |  |  |
| Events | 9,818 | 8,813 | 7,589 | 6,702 |  |
| Person-years | 568,280 | 600,513 | 600,356 | 608,596 |  |
| aHR (95% CI) | 1.00 (reference) | 0.91 (0.89-0.94) | 0.84 (0.81-0.86) | 0.76 (0.74-0.79) | <0.001 |
| Coronary heart disease |  |  |  |  |  |
| Events | 4,511 | 4,019 | 3,444 | 2,937 |  |
| Person-years | 568,280 | 600,513 | 600,356 | 608,596 |  |
| aHR (95% CI) | 1.00 (reference) | 0.88 (0.84-0.92) | 0.78 (0.75-0.82) | 0.67 (0.63-0.70) | <0.001 |
| Stroke |  |  |  |  |  |
| Events | 5,307 | 4,794 | 4,145 | 3,765 |  |
| Person-years | 568,280 | 600,513 | 600,356 | 608,596 |  |
| aHR (95% CI) | 1.00 (reference) | 0.94 (0.90-0.97) | 0.88 (0.85-0.92) | 0.85 (0.81-0.89) | <0.001 |
| **Modified modified continuity index, quartiles** | | | | |  |
|  | **1^st^ (lowest)** | **2^nd^** | **3^rd^** | **4^th^ (highest)** |  |
| Range | 0.24-0.78 | 0.78-0.84 | 0.84-0.89 | 0.90-1.00 |  |
| N | 61,027 | 60,805 | 61,299 | 61,056 |  |
| Cardiovascular disease |  |  |  |  |  |
| Events | 8,014 | 8,451 | 8,649 | 7,808 |  |
| Person-years | 603,960 | 591,266 | 590,073 | 592,348 |  |
| aHR (95% CI) | 1.00 (reference) | 0.94 (0.91-0.97) | 0.91 (0.89-0.94) | 0.85 (0.82-0.88) | <0.001 |
| Coronary heart disease |  |  |  |  |  |
| Events | 3,869 | 3,870 | 3,831 | 3,341 |  |
| Person-years | 603,960 | 591,266 | 590,073 | 592,348 |  |
| aHR (95% CI) | 1.00 (reference) | 0.92 (0.88-0.96) | 0.87 (0.83-0.81) | 0.77 (0.73-0.80) | <0.001 |
| Stroke |  |  |  |  |  |
| Events | 4,145 | 4,571 | 4,818 | 4,467 |  |
| Person-years | 603,960 | 591,266 | 590,073 | 592,348 |  |
| aHR (95% CI) | 1.00 (reference) | 0.96 (0.92-0.99) | 0.95 (0.92-0.99) | 0.93 (0.89-0.97) | <0.001 |

Hazard ratio calculated by Cox proportional hazards regression after adjustments for age, sex, household income, smoking status, alcohol intake, physical activitiy, Charlson comorbidity index, body mass index, and fasting serum glucose.

Acronyms: aHR, adjusted hazard ratio; CI, confidence interval.
